# Effects of rearing conditions on natal dispersal processes in a long‐lived predator bird

**DOI:** 10.1002/ece3.4165

**Published:** 2018-06-13

**Authors:** Maialen Azpillaga, Joan Real, Antonio Hernández‐Matías

**Affiliations:** ^1^ Departament de Biologia Evolutiva, Ecologia i CiènciesAmbientals del Institut de la Recerca de la Biodiversitat (IRBIO) Equip de Biologia de la Conservació Universitat de Barcelona Barcelona Spain; ^2^ Departament de Ciències Ambientals Facultat de Ciències Universitat de Girona Girona Spain

**Keywords:** body condition, Bonelli’s eagle, demographic heterogeneity, natal dispersal, raptor, rearing conditions

## Abstract

Natal or prebreeding dispersal is a key driver of the functioning, dynamics, and evolution of populations. Conditions experienced by individuals during development, that is, rearing conditions, may have serious consequences for the multiple components that shape natal dispersal processes. Rearing conditions vary as a result of differences in parental and environmental quality, and it has been shown that favorable rearing conditions are beneficial for individuals throughout their lives. However, the long‐term consequences of rearing conditions on natal dispersal are still not fully understood in long‐lived birds. In this study, we aim to test the following hypotheses to address the relationship between rearing conditions and certain components of the natal dispersal process in Bonelli’s eagle (*Aquila fasciata*): (1) The body condition of nestlings depends on the quality of the territory and/or breeders; and (2) the survival until recruitment, (3) the age of recruitment, and (4) the natal dispersal distance (NDD) all depend on rearing conditions. As expected, nestlings reared in territories with high past productivity of chicks had better body condition, which indicates that both body condition and past productivity reflect the rearing conditions under which chicks are raised. In addition, chicks raised in territories with high past productivity and with good body condition had greater chances of surviving until recruitment. Furthermore, birds that have better condition recruit earlier, and males recruit at a younger age than females. At last, although females in good body condition exhibited higher NDD when they recruited at younger ages, this pattern was not observed in either older females or males. Overall, this study provides evidence that rearing conditions have important long‐term consequences in long‐lived birds. On the basis of our results, we advocate that conservation managers work actively in the promotion of actions aimed at improving the rearing conditions under which individuals develop in threatened populations.

## 1 INTRODUCTION

Natal dispersal is a key driver of the functioning, dynamics, and evolution of populations and metapopulations (Clobert, Baguette, Benton, & Bullock, 2012; Debeffe et al., 2012; Dobson, 2012; Greenwood & Harvey, 1982). The natal dispersal process involves a complex sequence of behaviors including departure from the natal site, transience, and settlement or recruitment, although it is commonly summarized as the movement of an individual from site of birth to site of first reproduction (Clobert et al., 2012).

Variation in dispersal can to a large extent be explained by variation in the morphological, physiological, and behavioral traits that affect individual movement and orientation capacity (Clobert, Le Galliard, Cote, Meylan, & Massot, 2009). An individual’s internal state can provide information on the fitness potential of its environment, thereby affecting its decision to stay or leave its natal area (Clobert et al., 2012). In other words, the costs and benefits of natal dispersal are influenced by the internal state of individuals and by environmental conditions experienced both in the natal area and in future breeding sites (Acker et al., 2018; Bonte et al., 2012; Bowler & Benton, 2005; Clobert et al., 2009; del Mar Delgado, Penteriani, Revilla, & Nams, 2010; Rémy, Le Galliard, Gundersen, Steen, & Andreassen, 2011). Thus, both modeling and empirical studies indicate that natal dispersal behavior often represents a plastic‐, phenotype‐, and condition‐dependent strategy (Bonte et al., 2012; Clobert et al., 2012; Rémy et al., 2011).

Conditions experienced by individuals during their development—that is, their rearing conditions—may have serious consequences for fitness (Tilgar, Mänd, Kilgas, & Mägi, 2010). Thus, the understanding of this relationship is of major interest in the study of life history evolution, population ecology, and the interface between these two fields (Clobert et al., 2012; Rödel, Von Holst, & Kraus, 2009). In addition, an understanding of the impact of rearing conditions may be highly useful in management and conservation, yet the potential consequences for future survival of offspring reared under poor environmental conditions are usually ignored. Conditions during early life vary owing to differences in parental and environmental quality (Sergio et al., 2009; Van De Pol, Bruinzeel, Heg, Van Der Jeugd, & Verhulst, 2006), and a large body of evidence exists to show that favorable rearing conditions are beneficial for individuals, particularly in short‐term effects such as increased juvenile survival (Rémy et al., 2011; Rödel et al., 2009). In this sense, Green and Cockburn (2001) and Sergio et al. (2009) suggest that the probability of surviving to independence is closely related to a nestling’s own quality or body condition. In fact, the onset of dispersal is generally associated with high mortality rates due to predation, stress, energy depletion, and the lack of familiarity with novel environments (Hardouin et al., 2012). Thus, postfledging survival may be influenced by nestling mass because heavier individuals are fitter and so are better equipped to cope with short periods of food shortage (Green & Cockburn, 2001; Hsu, Dijkstra, & Groothuis, 2017), for example. As well, larger and heavier individuals may be dominant over smaller lighter ones and thus will have an advantage when attempting to disperse and/or recruit successfully (Cam & Aubry, 2011; Debeffe et al., 2012; Green & Cockburn, 2001; Hsu et al., 2017).

Less is known about the mid‐ and long‐term effects of rearing conditions on long‐lived species (Szostek & Becker, 2015), which include the complex set of processes that, together, conform natal dispersal, such as the probability of surviving until recruitment, that is, the entry of prebreeding birds into the breeding portion of the population (Green & Cockburn, 2001; Hernández‐Matías et al., 2010), the age at which individuals recruit (Acker et al., 2018; Grande et al., 2009; Hernández‐Matías, Real, Pradel, Ravayrol, Vincent‐Martin, 2011; Kokko & Sutherland, 1998), and the distance from the place of birth to the recruitment site, the so‐called natal dispersal distance (NDD) (Rémy et al., 2011; Stamps, 2006). This gap in our knowledge is understandable given that effects of rearing conditions are less pronounced during middle and late life stages (Van De Pol et al., 2006). In addition, individuals of long‐lived highly mobile species such as birds are difficult to monitor over the time and spatial spans on which their vital activity takes place (Kenward, Rushton, Perrins, Macdonald, & South, 2002; Newton, Mcgrady, & Oli, 2016). As a result, only long‐term studies based on the marking of large numbers of individually recognizable animals have ever been able to establish links between early life conditions and the long‐term consequences they may have on fitness components in long‐lived species (Carrete, Sánchez‐Zapata, Tella, Gil‐Sánchez, & Moleón, 2006; Hernández‐Matías et al., 2010; Verhulst, Perrins, & Riddington, 2011).

The factors determining recruitment processes in birds depend on individual and parental traits, and the particular characteristics of territories, years of birth, and years of recruitment (Cam & Aubry, 2011; Müller, Pasinelli, Schiegg, Spaar, & Jenni, 2005; Rutz & Bijlsma, 2006; Tella, Bortolotti, Dawson, & Forero, 2000). However, our understanding of the determinants of all these processes in long‐lived birds is poor, especially in territorial species (Hernández‐Matías et al., 2010). In many bird species, especially in long‐lived ones, individuals start breeding at different ages depending on their quality, a variation that, according to biological theory, results from a trade‐off between current and future prospects of survival and reproduction (Fay, Barbraud, Delord, & Weimerskirch, 2016; Stearns, 1992). In this regard, recruitment age can be explained by two different hypotheses: the late‐breeding and the early‐breeding hypotheses. The former posits that individuals delay breeding until they acquire the competitive abilities and the familiarity with resources they require to obtain a breeding site in the natal environment (Fasciolo, del Mar Delgado, Cortés, Soutullo, & Penteriani, 2016; Penteriani & Delgado, 2009; Serrano, Tella, Donázar, & Pomarol, 2003); thus, birds in better condition are expected to recruit later because their prospects of future survival are higher in this learning period. On the contrary, the latter hypothesis (redefined according to Serrano et al., 2003) suggests that competitiveness may be innate and not driven by experience; thus, more competitive individuals recruit earlier into the breeding population than less competitive ones. By recruiting earlier, individuals benefit from having the opportunity to begin their breeding careers sooner and thus improve their fitness (Fay et al., 2016; Hernández‐Matías, Real, Pradel, Ravayrol, et al., 2011; Mcgraw, Virginia, & Virginia, 1996; Oli, Hepp, & Kennamer, 2002). Birds will also gain more experience with their mate and/or knowledge of local features of the territory (Beletsky & Orians, 1991; Bradley, Wooller, Skira, & Serventy, 1990), which is also thought to improve their breeding performance over time (Hernández‐Matías, Real, Pradel, Ravayrol, et al., 2011).

Apart from age, spatial distance from the site of birth to the site of recruitment is an essential component of natal dispersal processes. Individuals raised under good conditions are more likely to stay in their natal environment due to a combination of low dispersal propensity and high competitive ability (Bowler & Benton, 2005; Clobert et al., 2012; Muriel, Morandini, Ferrer, Balbontín, & Morlanes, 2016). On the other hand, high‐quality individuals may also be better competitors and/or be better able to bear the costs of dispersal, in which case the quality of the rearing environment might in fact have a positive rather than negative effect on dispersal (Bonte et al., 2012; Bowler & Benton, 2005; Clobert et al., 2012; Debeffe et al., 2012). So, as long‐distance dispersal is energetically expensive and may involve a high mortality risk, the acquisition of a good body condition prior to dispersal plays a critical role in determining the extent to which animals disperse (Barbraud, Johnson, & Bertault, 2003). As a consequence, a disperser with large energy stores might be able to assess more potential breeding habitats before running out of energy than a disperser with fewer energy resources (Barbraud et al., 2003; Stamps, 2006; Tilgar et al., 2010). Also, dispersal can produce net benefits including the avoidance of inbreeding and the reduction of competition for resources and mates; although the distances required to avoid inbreeding are likely to differ from those required to escape resource competition (Bowler & Benton, 2005).

In this study, we test six hypotheses related to several key components of the relationship between rearing conditions and the natal dispersal process in a long‐lived territorial bird, Bonelli’s eagle *Aquila fasciata* (see Table 1). First, we test the hypothesis that body condition in nestlings depends on the quality of territory and/or breeders (Hypothesis 1). We assume that both the quality of territories and the body condition of chicks reflect to some extent the conditions under which chicks are reared and, based on this, we predict that individuals raised in better quality territories will have better body condition. Second, we test whether the probability of surviving until territorial recruitment depends on rearing conditions (Hypothesis 2) and predict that individuals in better body condition will have an advantage in terms of survival until territorial recruitment. Third, we study whether the age of recruitment depends on rearing conditions and test two complementary hypothesis described above: the late‐breeding (Hypothesis 3) and the early‐breeding hypotheses (Hypothesis 4). According to Hypothesis 3, we would expect individuals in better body condition to recruit later; by contrast, Hypothesis 4 predicts that individuals in better body condition recruit at a younger age. Fourth, we study whether natal dispersal distance depends on rearing conditions and test two further hypotheses. Hypothesis 5 assumes that dispersal is costly and so recruiting at the natal site or at shorter distances will be the best strategy; hence, we predict that individuals in better body condition recruit at shorter distances. On the other hand, Hypothesis 6 assumes that natal dispersal can produce net benefits including the avoidance of inbreeding and the reduction of competition for resources and mates and, accordingly, we predict that individuals in better body condition recruit farther from their natal territories.

**Table 1:** Description of the main hypotheses studied, the predictions expected for each according to general theories of territorial birds, and the factors considered for their analysis in this study

Factor	Hypothesis	Prediction
Body condition	1. Body condition in nestlings depends on the quality of territories and/or breeders	Nestling’s body condition increases with the quality of the breeding territory and/or breeders
Survival	2. Survival until recruitment of individuals depends on their body condition during development	Chicks in better body condition will have an advantage in terms of survival until territorial recruitment
Age of recruitment	3. Late‐breeding	Individuals in better body condition recruit later
	4. Early‐breeding	Individuals in better body condition recruit earlier
Natal dispersal distance	5. Individuals avoid costs associated to disperse at larger distances	Shorter natal dispersal distance in individuals in better body condition
	6. Individuals avoid costs associated to disperse at shorter distances	Larger natal dispersal distance in individuals in better body condition

## 2 MATERIALS AND METHODS

### 2.1 Study species and target population

Bonelli’s eagle (*Aquila fasciata*) is a territorial accipitrid that is irregularly distributed from Southeast Asia through the Middle East to the western Mediterranean (del Hoyo, Elliott, & Sargatal, 1994). Its European population has been estimated at 920–1,100 pairs, of which c. 80% are found in the Iberian Peninsula (BirdLife International 2004; del Moral, 2006). This species has undergone a dramatic decline in recent decades and is now listed as endangered in Europe (2009/147/EC; BirdLife International 2004).

Like other eagle species, Bonelli’s eagle is long‐lived and monogamous, and exhibits delayed maturity, small clutch size, and low annual productivity (del Hoyo et al., 1994; Real & Mañosa, 1997). Bonelli’s eagle populations are composed of two fractions, nonterritorial and territorial birds that have markedly different lifestyles. Once the dependence period ends, Bonelli’s eagles enter a transient nomadic phase or dispersal period during which they perform long‐distance movements to dispersal areas (i.e., areas characterized by high prey abundance where nonadult individuals temporarily settle before recruitment) and show no territorial behavior (Cadahía, López‐López, Urios, & Negro, 2010; Real & Mañosa, 2001; Soutullo, López‐López, Cortés, Urios, & Ferrer, 2013). This period before recruiting into a territory may last for several years (Real & Mañosa, 2001) and typically ends by the third or fourth years of life (Hernández‐Matías et al., 2010). By contrast, territorial Bonelli’s eagles are sedentary and have strong site fidelity (Hernández‐Matías, Real, Parés, & Pradel, 2015). Here, we use a broad definition of natal dispersal that encompasses several processes that shape the life stage lasting from the end of parental dependence to territorial recruitment, which includes survival until recruitment, the recruitment age, and the distance from natal to recruitment territories.

We studied a Bonelli’s eagle population in Catalonia (NE Iberian Peninsula), an area characterized by habitats containing Mediterranean landscape features, an average annual rainfall of 425–664 mm, and where nesting areas are situated at 30–1,200 m a.s.l. The study population consisted of 85–90 pairs during the 1970s but decreased in number until it stabilized at 63 pairs in 2000; nevertheless, in recent years, numbers have risen to 73 pairs (DTS 2016; Real, Tintó, Boran, Beneyto, & Parellada, 2004). However, this increase is not a response to improvements in the principal vital rates—which have in fact worsened in recent years—but are, rather, the consequence of the net entry of immigrant eagles from neighboring populations (Hernández‐Matías et al., 2013, 2015).

### 2.2 Field procedures

**Figure 1:**
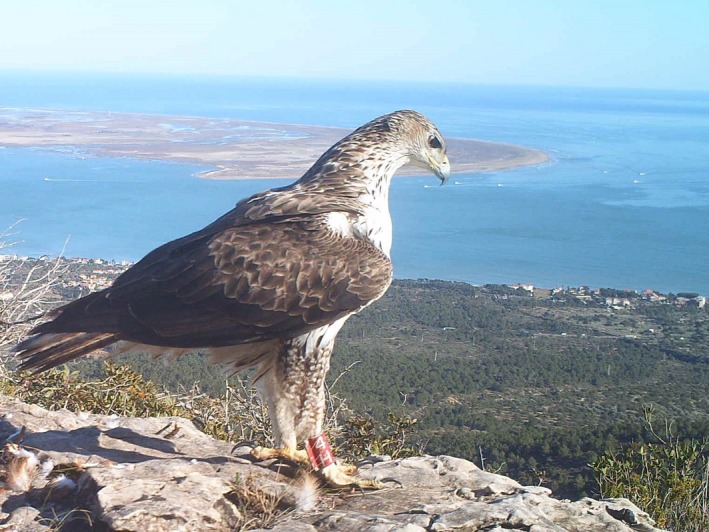
Adult Bonelli’s eagle. Picture authorship: Equip de Biologia de la Conservació—Universitat de Barcelona

The study population was intensively monitored from 1998 to 2016. Monitoring was performed in c. 70% of the population’s territories via repeated visits to breeding areas in January–July (minimum three visits) that gathered information regarding the occupation status of territories, individual identity (if ringed), plumage‐age, sex of territorial birds, and the number of fledged chicks. A representative sample of nestlings has been ringed annually in the study population since 2008 (*n* = 340; c. 70% of fledged chicks; Figure 1). Once nestlings are 35–45 days old, with the aid of experienced climbers, chicks are fitted with alphanumerically coded colored metal rings that allow individual identification at distance. In addition, the following measurements are taken: weight, measured with a spring balance to the nearest 25 g; tarsus length from the back of the tarsal joint to the front of the folded central toe; anteroposterior and transversal tarsus diameter at the middle point of the leg; culmen length from bill tip to the distal edge of the nostril; claw length and central toe nail length, measured dorsally from the base to the tip of the claw; and foot length measured ventrally with the foot resting on a flat surface, from the base of the central nail to the base of the claw. All these measurements were taken with a digital caliper to the nearest 0.01 mm. The seventh primary and the central tail feather length were measured with a metal ruler to the nearest 1 mm from the tip of the feather to the skin insertion point. Sex, age, and the number of chicks in each nest were also recorded. Sex was determined using a discriminant function estimated from a subsample of 190 chicks sexed by DNA following the method described in Fridolfsson and Ellegren (1999).

Further visits to territories enabled us to monitor the recruitment of marked birds. The age of individuals at the time of recruitment was taken to be the number of calendar years between birth and the first time the bird was detected as territorial. Given that nonadult individuals sometimes return to breeding areas (but not to breed), a bird was considered to be recruited if it was observed on several occasions in the same year as one of a pair exhibiting territorial behavior such as roosting in the breeding area, nest‐building, and courting (Hernández‐Matías et al., 2010). In addition, populations around the focal area in Catalonia (Aragon and, specially, Castelló and France) are also intensively monitored; since similar schemes of ringing and monitoring are carried out on these regions. Indeed, some of the chicks born in the Catalonia population were observed to recruit in these neighboring populations.

For a summary of the data set resulting from this work and used in this study see Supplementary Information Table S1.

### 2.3 Estimation of rearing conditions

Conditions under which individuals develop may have strong consequences for their future performance (Rödel et al., 2009; Van De Pol et al., 2006). However, measuring these so‐called rearing conditions is challenging, particularly in wild animals, as they may depend on a large set of factors interacting in a complex fashion (e.g., parental care, feeding rates, food quality, and exposure to diseases). Here, two measures were regarded as proxies of rearing conditions: the past breeding performance or past productivity of the territory, and the body condition index of chicks. The former reflects the general rearing conditions experienced in the territory in question, while the latter measures the overall physical condition of chicks during the prefledgling stage.

In Bonelli’s eagle, the location of territories is relatively stable over time and most breeding areas remain unchanged for decades. In addition, territorial individuals have great fidelity to their territories, which means that pairs stay for years in the same area and attempt to breed annually. Consequently, the number of chicks fledged by a pair in a given year will reflect the quality of the two members of the pair and the environmental conditions that operate during the breeding season. Yearly conditions, though, are subject to environmental stochasticity, and thus, the average number of fledged chicks over a longer period will be a better reflection of the overall quality of the parents and environmental conditions that characterize the territory. Therefore, the average productivity of the territory in question over the past 10 years (referred to as “past productivity” in the analyses) was considered as a proxy of the rearing conditions experienced by chicks in that territory (mean = 1.13; range = 0–2; *n* = 340).

Body condition is a key characteristic of organisms that is commonly used in animal biology to quantify the health and physiological state of individuals (Labocha & Hayes, 2012; Stevenson & Woods, 2006). The use of morphological indices is commonplace when assessing animal body condition (Stevenson & Woods, 2006) but is not exempt from controversy (Peig & Green, 2009). A major drawback with calculating body condition indirectly via morphological measurements lies in the general problem of relative growth. The absolute size of fat stores or protein (typical measurements of fitness) tends to covary with body size, and so larger animals will have more absolute fat and will consequently be in better condition according to estimations. Therefore, a major challenge of a condition index is to control for growth effects in body size and, implicitly, the size of distinct body components.

Here, residuals of body mass regressed on a structural measure of body size were considered as a body condition index (Labocha & Hayes, 2012). Although this general method has been widely used in previous studies (Labocha & Hayes, 2012; Labocha, Schutz, & Hayes, 2014), we applied the procedures used in morphometric analysis to remove the size signal in the study of allometric processes.

First, we log‐transformed all recorded length measures of the chicks and then standardized the data to give a mean of 0 and a *SD* of 1. As the study species shows marked sexual dimorphism, standardization was done separately for males and females. Afterward, we pooled the two subsets to produce a single data set.

Body weight was considered as a representation of body mass. Body size was estimated from the scores on the first axis (PC1, hereafter “body size”) of a principal components analysis (PCA) of the covariance matrix built using tarsus, claw, seventh primary feather, and central tail feather length. These traits were selected because they gave the highest loadings in the PCA and therefore are the traits that best contribute to explaining size variation.

Next, we performed a linear model with body weight as dependent variable and body size as independent variable. Initially, we included the effect of sex and its interaction with body size to exclude the possibility that there were any allometric differences between sexes; however, after checking that these effects were not relevant we only considered body size as an independent variable (Supplementary Information Table S2). The residuals from the fitted model were taken as a measure of the body condition of each nestling. We validated these values by checking that they did not show any strong correlation with the chosen size variables, which meant that the size effect had been successfully removed (Supplementary Information Table S3).

### 2.4 Natal dispersal distance

Natal dispersal distances (NDD) were calculated as the Euclidean distance from the site of birth to the site of recruitment (km).

### 2.5 Statistical analysis

All our hypotheses were tested by building generalized linear mixed models (GLMM) using the *lme4* R package (Bates, Maechler, & Bolker, 2012), with the most suitable link function and error distribution for each analysis. Our general approach consisted in keeping the analyses as simple as possible, meaning that we only considered in our models those variables strictly necessary to test the addressed hypothesis. Nevertheless, there were some variables or effects that could potentially confound our results or behave as sources of nonindependence. In this sense, year and territory were considered as random factors in all the analyses, in order to account for the potential nonindependence of clustered observations. In addition, prior to the analysis in question, we analyzed the effects of random factors by comparing fully null models with models only considering the random factors (Supplementary Information Table S4). The fact that the variance explained by territory was much higher than that explained by year prevented us to consider the random effect of territory nested by year. Our aim was to assess the effect on the response variable of latent effects associated with year or territory and not explicitly considered in our analyses. In addition, we included in the analyses some variables that, despite not being directly related to the hypotheses we tested, previous knowledge suggests may have a relevant effect on the response variables studied (see below and Table 2 for a summary of all the full models fitted).

**Table 2:** Summary of all the full models fitted for the different hypotheses tested. The effects of territory and year were accounted for in all models by considering them as random factors

Hypotheses	Dependent variable	Model
1.	Body condition	Past_Productivity + Num_Nestlings + Past_Productivity*Num_Nestlings
2.	Survival until recruitment	Body_Condition + Past_Productivity + Age
3. and 4.	Age of recruitment	Body_Condition + Past_Productivity + Sex + Body_Condition*Sex + Body_Condition*Past Productivity + Past_Productivity*Sex
5. and 6.	NDD	Age_Recruitment + Body_Condition + Past_Productivity + Age_Recruitment*Body_Condition

To test hypothesis 1, body condition of chicks (dependent variable) was modeled as a normal response variable using the identity link function and assuming the error was normally distributed (*n* = 340). We considered past productivity, the number of chicks in each nest, and their interactions as explanatory variables. The number of reared chicks was included because it has been shown to have an effect on the body condition of nestlings in several long‐lived bird species (Monaghan, 2008).

To test hypothesis 2, survival until recruitment (the fledgling was either recruited or not) was chosen as the dependent variable and modeled as a binomial response variable using the logit link function, assuming the error to be binomially distributed (*n* = 340). In fact, even though some of the chicks born in our population recruited on neighboring regions, 85% of chicks recruited in the first 200 km around the natal nest. As a result, even the chicks born in the edges of our study area are very likely to be detected elsewhere once they recruit. In this analysis, past productivity, body condition, and the current age of the individual were fitted as explanatory variables. The age of the individual was considered in the analysis to account for the fact that young individuals are less likely to be recruited (Hernández‐Matías et al., 2010).

To test hypotheses 3 and 4, we modeled the age of recruitment (dependent variable) using the logarithm as a link function and the Poisson error distribution (*n* = 62). We fitted past productivity, body condition, and sex of each nestling and their interactions as explanatory variables. Sex was included in this analysis because previous studies indicate that in this population, females tend to recruit later than males (Browne, M., Real, J., Ponchon, C., Ravayrol, A., & Hernández‐Matías, A., unpublished data). For this and the following hypothesis tests, we used a smaller data set that only contained the individuals that had achieved territorial recruitment (62 out of 340).

**Figure 2:**
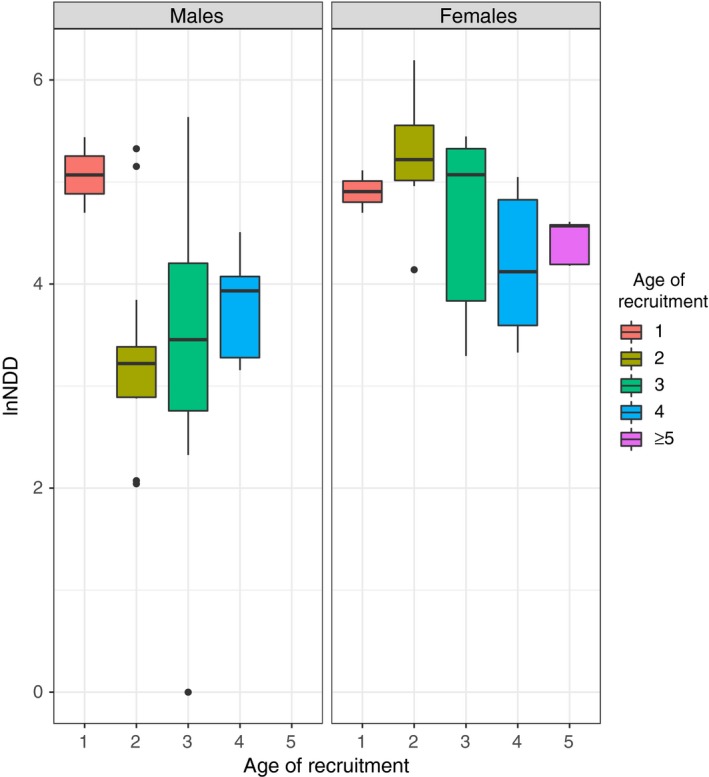
Effect of the age of recruitment on the NDD of males and females (*n* = 31 and *n* = 31, respectively)

At last, to test hypotheses 5 and 6, we first log‐transformed NDD in order to normalize this variable. Next, lnNDD (dependent variable) was modeled as a normal response variable using the identity link function and assuming the error to be normally distributed (*n*: Males = 31; Females = 31). We considered age of recruitment, past productivity, body condition, and the interaction between age of recruitment and body condition as explanatory variables. Although statistical analyses showed that the interaction between sex and age of recruitment did not have a statistically significant effect on NDD (Supplementary Information Table S5), previous knowledge (Browne, M., Real, J., Ponchon, C., Ravayrol, A., & Hernández‐Matías, A., unpublished data) as well as the complex patterns detected in the exploratory graphical analysis of the data (Figure 2) advised us to analyze males and females separately (see Supplementary Information Table S6 for an analysis of both sexes together). In fact, there is general evidence that sex is an important source of variation in dispersal among birds (Barbraud et al., 2003). In particular, female‐biased dispersal has been a common finding among many species (Bowler & Benton, 2005; Tilgar et al., 2010), especially in territorial dimorphic birds (Acker et al., 2018; Balbontín & Ferrer, 2009; Hernández‐Matías et al., 2010; Soutullo, Urios, Ferrer, & Peñarrubia, 2006).

Collinearity of explanatory variables was assessed by means of the analysis of variance inflation factors (VIF) and, if necessary, variables showing VIF values greater than 3 were excluded from the analyses (Zuur, Ieno, & Elphick, 2010). For model selection, we fitted several models considering all potential combinations of the principal effects of the explanatory variables and included some interactions that were considered biologically relevant in the context of the hypotheses tested. Model comparison and selection was performed using Akaike’s information criterion corrected for small sample sizes (AICc), the models with the smallest AICc value being best supported (Burnham & Anderson, 2002). We also estimated the Akaike weights (*w*_*i*_) of each model as a measurement of model plausibility. In addition, we estimated the parameters of an average model by taking into account those models that had a ΔAICc value less than 2 (Burnham & Anderson, 2002) in relation to the best‐fitted model. This procedure allows us to account for model selection uncertainty and to obtain more robust parameter estimates and predictions. At last, we calculated the sum of the Akaike weights of the selected variables for the average model as a measure of their relative importance for explaining the variance of the model (Grueber, Nakagawa, Laws, & Jamieson, 2011).

All statistical analyses were performed using R statistical software v.3.3.3 GUI 1.69 Mavericks build (R Core Team 2017); all plots were performed using the *ggplot2* R package (Wickham & Chang, 2016).

## 3 RESULTS

### 3.1 Relationship between territory quality and chick body condition

The analysis of the null model and the models considering random factors revealed that the effect of territory and year was important in the body condition (*n* = 340; Supplementary Information Table S4).

**Figure 3:**
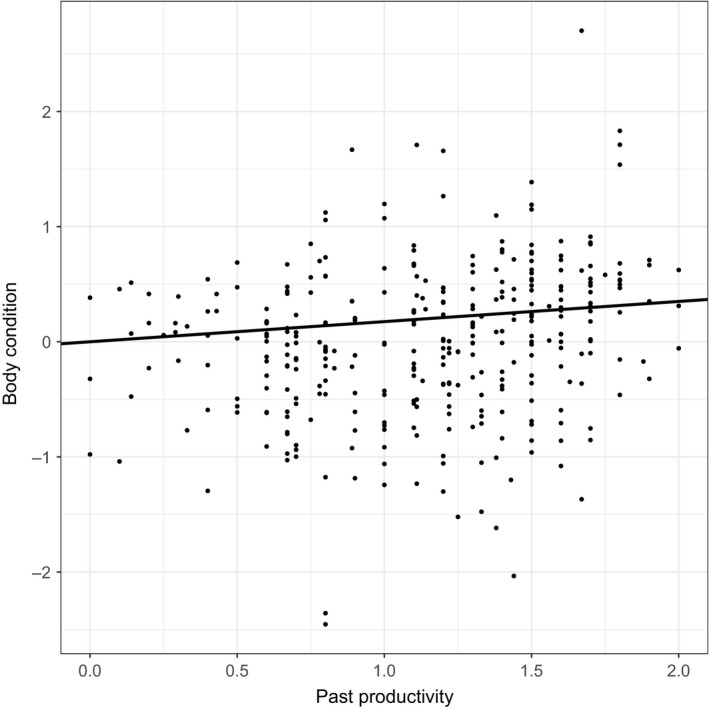
Effect of the past productivity of territories on chick body condition (*n* = 340). Body condition corresponds to the residuals of the regression of body size on body mass for each nestling (see text for details). Dots illustrate the observed values for body condition of chicks. The line illustrates the predicted values of body condition estimated from the average model for the observed range of past productivity values

Once the independent variables were considered, we selected the two best‐fitted models with a ΔAICc < 2 out of the eight models fitted (Supplementary Information Table S7) for the average model construction (Table 3). Body condition of nestlings increased with greater values for past productivity (Coefficient = 0.25; *SE* = 0.10), this variable being the most important predictor of body condition (Table 3; Figure 3). In addition, the number of nestlings in each nest had a negative effect on body condition (Coefficient = −0.03; *SE* = 0.08), although, as illustrated by the confidence interval including the 0 value, this effect was very weak.

**Table 3:** Effects on body condition of the parameters selected from the best‐fitting models (ΔAICc < 2) after model averaging. The relative importance of considered variables expresses the sum of the Akaike weights of models containing the parameter in question. The effects of territory and year were accounted for in all the models by considering them as random factors

Parameter	Estimate	*SE*	95% Confidence interval	Relative importance
Intercept	−0.279	0.137	(−0.548, −0.009)	
Past_productivity	0.250	0.101	(0.051, 0.450)	1.00
Num_Nestlings	−0.030	0.079	(−0.185, 0.125)	0.28

As shown in Figure 3, the territories with a high past productivity produced chicks in relatively good body condition, while chicks from territories with a lower past productivity were in poorer body condition. Nonetheless, heterogeneous patterns occur in territories with a medium past productivity value, where territories with a similar past productivity value produce chicks in either good or poor body condition.

### 3.2 Effects of rearing conditions on the probability of survival until recruitment

The analysis of the null model and the models considering random factors also revealed that the effect of territory and year was important in the survival of fledglings until they achieved territorial recruitment (*n* = 340; Supplementary Information Table S4).

**Figure 4:**
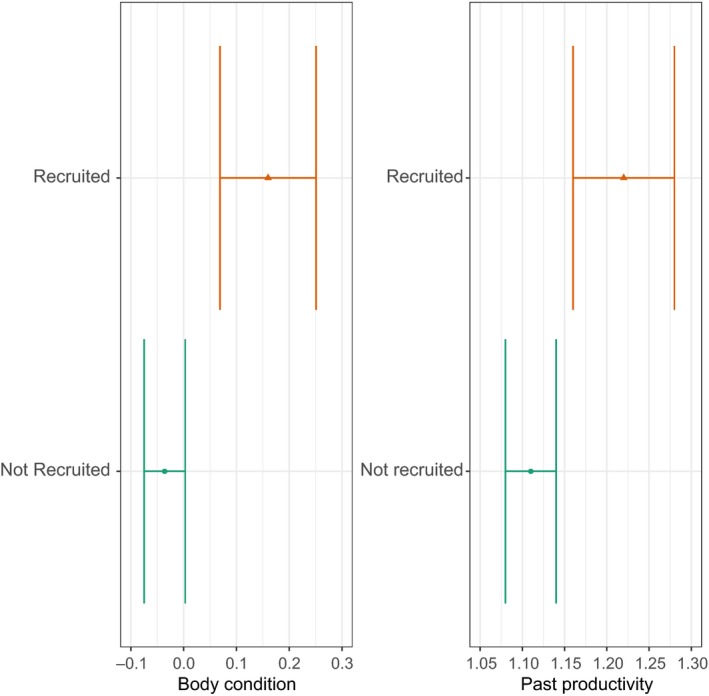
Effects of body condition (left) and past productivity of the territory (right) on the survival probability (*n* = 340). Dots illustrate the mean of the body condition and the past productivity of the territories, respectively, of nestlings that have (orange) and have not (green) been recruited. Bars represent their associated standard errors

**Figure 5:**
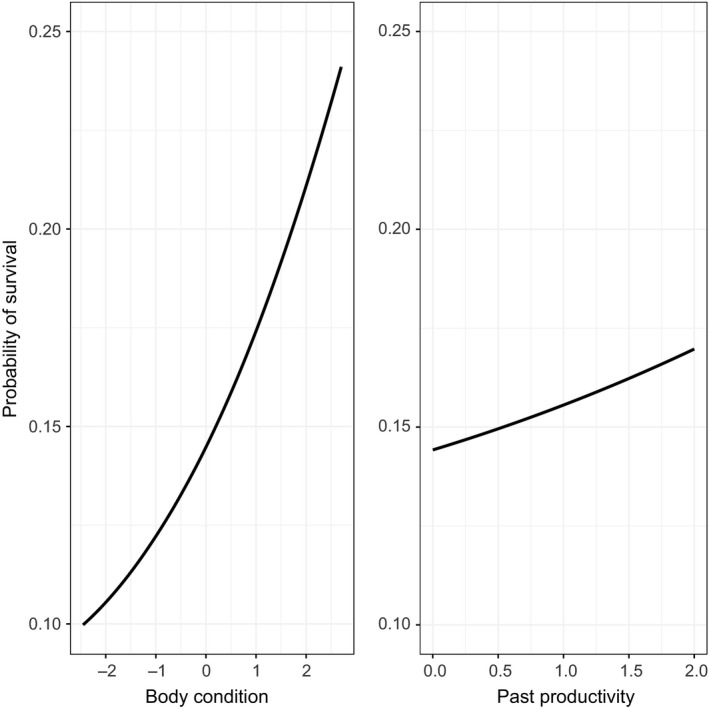
Predicted effects of body condition (left) and past productivity of the territory (right) on the probability of survival until recruitment (*n* = 340) based on the average model and assumed the average age at which birds were observed to recruit (2.95 years)

Eight models were fitted for this analysis, of which we selected the best four models to construct the average model (Supplementary Information Table S8). The age of each bird, its body condition, and the past productivity of its natal territory were the three variables included in the selected models. All these variables had positive coefficients showing that survival increased in chicks with good body condition that were raised in territories with high past productivity (Figures 4 and 5). The current age of individuals was the most important predictor of the probability of survival, which indicates that very young individuals are not likely to be already recruited (Table 4).

**Table 4:** Effects on survival until recruitment of the parameters selected from the best‐fitting models (ΔAICc < 2) after model averaging. The relative importance of considered variables expresses the sum of the Akaike weights of the models containing the parameter in question. The effects of territory and year were accounted for in all the models by considering them as random factors

Parameter	Estimate	*SE*	95% Confidence interval	Relative importance
Intercept	−0.804	0.546	(−3.878, −1.731)	
Age	0.196	0.073	(0.052, 0.340)	1.00
Body_condition	0.379	0.243	(−0.099, 0.856)	0.55
Past_productivity	0.315	0.418	(−0.507, 1.137)	0.32

### 3.3 Effects of rearing conditions on recruitment age

The effect of territory or year on the age of recruitment was weak (*n* = 62; Supplementary Information Table S4) in this case.

**Figure 6:**
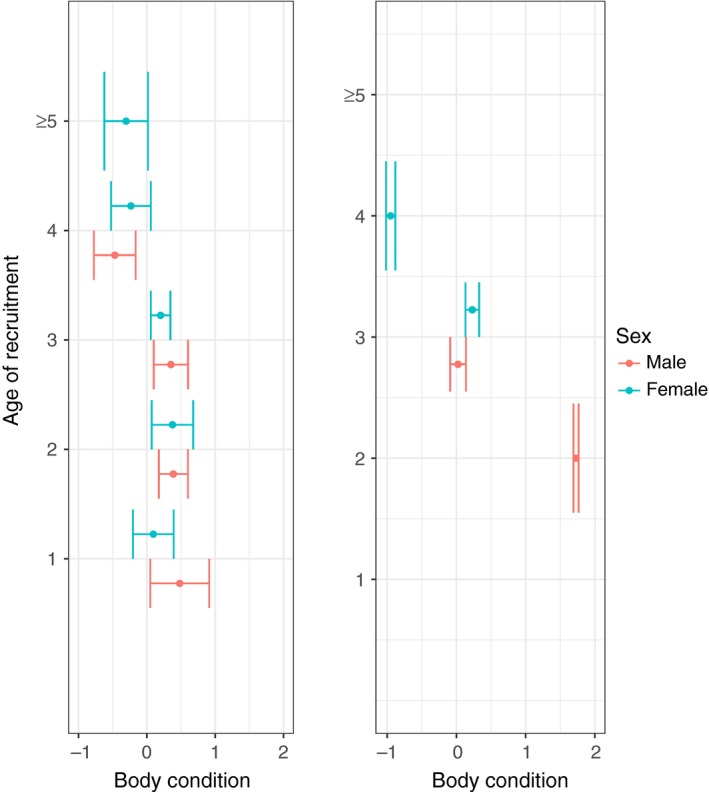
Left: Body condition for recruited male (red) and female (blue) Bonelli’s eagles for each age of recruitment (*n* = 62; males = 31 and females = 31). Body condition corresponds to the residuals of the regression of body size on body mass for each nestling (see text for details). Dots illustrate the mean of the body condition of chicks recruited in each age and bars their associated standard errors. Right: Effects of body condition on the recruitment age of male (red) and female (blue) Bonelli’s eagles. Dots illustrate the mean predicted recruitment age values estimated from the average model for different values of body condition

Six models that included body condition, sex, and past productivity as explanatory variables were selected out of the 64 models evaluated (Supplementary Information Table S9). Body condition and past productivity had a negative coefficient (coefficient = −0.20; *SE* = 0.11 and coefficient = −0.18; *SE* = 0.18, respectively), unlike sex, which had a positive coefficient (coefficient = 0.21; *SE* = 0.15). Based on the relative importance parameter, body condition was the most important predictor followed by sex and past productivity (see Table 5). Overall, males recruited at younger ages than females (Figure 6).

**Table 5:** Effects on age of recruitment of the parameters selected from the best‐fitting models (ΔAICc < 2) after model averaging. The relative importance of considered variables expresses the sum of the Akaike weights of the models containing the parameter in question. The effects of territory and year were accounted for in all the models by considering them as random factors

Parameter	Estimate	*SE*	95% confidence interval	Relative importance
Intercept	1.031	0.282	(0.474, 1.587)	
Body_condition	−0.197	0.111	(−0.418, 0.025)	0.61
Sex	0.213	0.150	(−0.087, 0.513)	0.36
Past_productivity	−0.184	0.177	(−0.538, 0.171)	0.23

### 3.4 Effects of rearing conditions on natal dispersal distance

In both males and females, the effect of territory or year on the NDD appeared to be weak (*n* = 31 and *n* = 31, respectively; Supplementary Information Table S4).

#### 3.4.1 Males

Twelve models were fitted for the NDD for males, of which the null model was the best‐fitted, with more than two ΔAICc points from the next model (Supplementary Information Table S10). Thus, in the case of males, none of the explanatory variables considered allows us to explain the variation in NDD. Despite this, a graphical representation of the data illustrates that, with the exception of the chicks recruited in the first year, younger males tend to recruit closer to their natal territory than older ones (Figure 2).

#### 3.4.2 Females

Four models including body condition, age of recruitment, and their interaction as explanatory variables were selected from the twelve evaluated models (Supplementary Information Table S11). Age of recruitment and its interaction with body condition had a negative coefficient (coefficient = −0.17; *SE* = 0.09 and coefficient = −0.36; *SE* = 0.12, respectively), unlike body condition, which had a positive coefficient (Coefficient = 1.05; *SE* = 0.64). Based on the relative importance parameter, body condition was the most important predictor, followed by age of recruitment and the interaction between these two variables (Table 6). Nevertheless, this last variable was the only one not to contain the zero value in its confidence interval.

**Table 6:** Effects on female NDD of the parameters selected from the best‐fitting models (ΔAICc < 2) after model averaging. The relative importance of considered variables expresses the sum of the Akaike weights of the models containing the parameter in question. The effects of territory and year were accounted for in all the models by considering them as random factors

Parameter	Estimate	*SE*	95% confidence interval	Relative importance
Intercept	4.825	0.368	(4.084, 5.565)	
Body_Condition	1.047	0.639	(−0.225, 2.320)	0.55
Age_Recruitment	−0.174	0.093	(−0.365, 0.018)	0.53
Age_Recruitment*Body_Condition	−0.360	0.116	(−0.600, −0.119)	0.37

**Figure 7:**
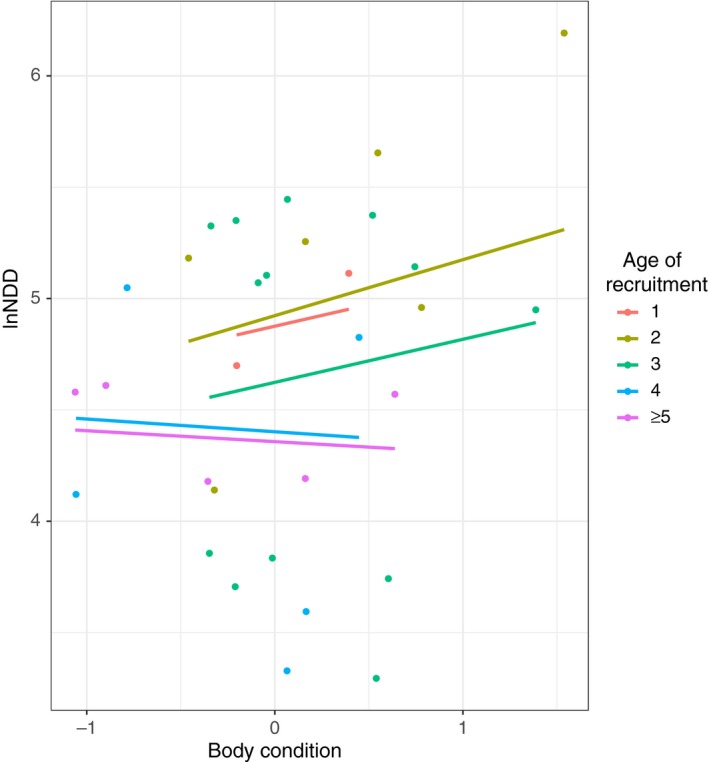
Effects of body condition and age of recruitment on the NDD performed by females (*n* = 31). Dots illustrate the observed NDD for each value of body condition. Lines illustrate the predicted NDD estimated from the average model for different values of body condition and recruitment ages

Thus, as shown in Figure 7, NDD increases with body condition in the youngest females (ages 1, 2, and 3), an effect that is not present in older females. In addition, younger females tend to recruit at greater NDD than older ones (Figures 2 and 7).

## 4 DISCUSSION

This study illustrates the fact that the rearing conditions under which Bonelli’s eagle nestlings develop have strong consequences on relevant components of the natal dispersal process (Cam & Aubry, 2011; Rödel et al., 2009; Van De Pol et al., 2006), such as the survival until recruitment, the recruitment age, and the natal dispersal distance. A detailed study of natal dispersal was made possible by intensive long‐term monitoring of the study species performed over a large geographical area that included the ringing of nestlings, the taking of morphometric measurements, and the recording of survival, reproduction, and recruitment information, as well as the use of generalized linear mixed models to test several relevant hypotheses on this topic (see Table 7 for a summary of our results). Our findings may also prove to be useful for improving management and conservation actions designed to improve the conservation status of endangered species.

**Table 7:** Description of the support for the observed results given the considered hypotheses. “F” illustrates full support, “P” illustrates partial support, and “N” illustrates no support for the hypotheses

Factor	Hypothesis		Results support	
Body condition	1.	Better performing territories/progenitors improve body condition	F	
Survival until recruitment	2.	Body condition increases survival until recruitment	F	
Age of recruitment	3.	Late‐breeding	N	
	4.	Early‐breeding	F	
Natal dispersal distance	5.	Body condition decreases distance	Males	P
			Females	P
	6.	Body condition increases distance	Males	P
			Females	P

### 4.1 Relationship between territory quality and chick body condition

The observed effects of the natal territory and the year of birth on the body condition of the chicks support the idea that marked spatial and temporal environmental heterogeneity exists in the factors that determine rearing conditions. Spatial environmental heterogeneity may also be a consequence of how local sites differ in their suitability for survival and reproduction (Carrete et al., 2006). Interestingly, the productivity of territories may also shift over time as territories vary due to changes in environmental conditions (i.e., quantity/quality of prey, inter‐ and intraspecific competition, climate change, etc.).

The positive relationship found between body condition of chicks and past productivity of territories suggests that our initial assumption—that these two variables reflect the rearing conditions under which chicks develop—is correct. In addition, this relationship underscores the fact that the prospects of being reared in good conditions are not homogeneous, and that high‐quality territories and parents produce nestlings with better body condition. This pattern is explainable by the fact that the best territories usually have more abundant and readily accessible food—the early development of chicks depends heavily on food provisioning by both parents (Rödel et al., 2009; Sergio et al., 2009)—and high‐quality individuals tend to occupy high‐quality territories (Ferrer & Bisson, 2003).

The presence of siblings is an important component of the early developmental environment in most mammal and bird species (Rödel et al., 2009) and may explain the negative relationship observed between body condition and the number of siblings in the nest. In fact, several studies of mammals and birds have shown that parents cannot fully compensate for the increased energetic requirement of an enlarged brood (Monaghan, 2008; Rémy et al., 2011). Bonelli’s eagles usually lay one or two eggs (Real, 1991), so females have to face a trade‐off between forming one or two eggs at the time of egg formation. If two chicks survive, parental care (e.g., brooding and food provisioning) will be substantially more intensive. Later, environmental conditions may change during the breeding season and the clutch size may not be optimal given the novel conditions (Bonte et al., 2012). Furthermore, given that Bonelli’s eagle is a long‐lived species, the parents’ priority is to keep their own survival possibilities high and their required reproductive effort low (Hernández‐Matías, Real, Pradel, Ravayrol, et al., 2011). Thus, the effort required to rear two chicks under optimal conditions may not be rewarded and chicks may fledge with poorer body conditions or even die (Hernández‐Matías, Real, Parés, & Llacuna, 2016). In addition, the hatching order of chicks may also have an effect on the body condition of nestlings as has been observed in many bird species such as common kestrels *Falco tinnunculus* (Martínez‐Padilla, Vergara, & Fargallo, 2017) or Kittiwakes *Rissa tridactyla* (Cam, Monnat, & Hines, 2003), among others. However, given the difficulty to stablish the nestling’s rank in many of the studied broods, we could not address this issue in the present study.

It is noticeable that in the studied population, there are a number of heterogeneous patterns in the relationship between body condition and the past productivity of the territory. In fact, while chicks from territories with a high past productivity have quite good body condition, territories with a middle past productivity value produce chicks in either good or poor body condition. This pattern might be explained because temporal variation in environmental variables might differ between these territories. Otherwise, it may be because the territories with nestlings in good body condition are territories rich in prey quality/quantity but with high rate of replacements (i.e., high adult mortality). In turn, territories with nestlings in bad body condition may be of low quality due to the poor quality/low quantity of prey or other negative environmental factors (e.g., human disturbance). In this regard, Penteriani, Balbontin, and Ferrer (2003) observed that more than 60% of recorded causes of breeding failure in Bonelli’s eagles were related to human presence and disturbance. Therefore, our results suggest that the body condition of chicks may provide a more accurate measurement of breeding condition than the number of fledged chicks.

### 4.2 Effects of rearing conditions on survival until recruitment

The factors determining recruitment processes in birds may depend on rearing conditions and on the characteristics of territories and years of both birth and of recruitment (Cam & Aubry, 2011; Hernández‐Matías et al., 2010). Here, we show that both body condition and territory quality (inferred from past reproduction success) have additive effects on the survival of fledglings until recruitment.

In long‐lived species, survival rates during lifespans are expected to follow a nonlinear pattern and are determined by a variety of constraints and selective pressures (Grande et al., 2009; Hernández‐Matías, Real, Pradel, Ravayrol, et al., 2011; Stearns, 1992). In territorial raptors, one of the most critical stages occurs when the parents expel their fledglings from their natal territory and young birds enter into a new transient nomadic phase—the dispersal period—which in Bonelli’s eagles implies long‐distance movements to dispersal areas and no territorial behavior (Cadahía et al., 2010; Real & Mañosa, 2001). Given that in our case, both territory quality and chick body condition are correlated, an explanation for our results might be that individuals with better body condition are better equipped to cope with short periods of food scarcity (Green & Cockburn, 2001; Hsu et al., 2017); alternatively, the greater concentrations of trophic resources near better territories may allow juveniles to improve hunting skills before leaving their natal territory (Sergio et al., 2009). However, the spatial and behavioral ecology of floaters (i.e., birds that have already dispersed from their natal territories) is still poorly understood, and thus, it is hard to assess how the variety of factors that come into play on nonbreeding grounds shape their survival (Barbraud et al., 2003; Bonte et al., 2012; Grande et al., 2009; Penteriani, Delgado, & Campioni, 2015). The fact that we found a measurable signal of the effects of rearing conditions on the probability of surviving until recruitment indicates that conditions in the early lives of individuals may have severe long‐term consequences on future fitness (Hsu et al., 2017). Body condition may also reflect the quality of individuals, which would imply that survival rates are shaped by the individual quality of birds (Barbraud et al., 2003; Green & Cockburn, 2001; Saunders, Arnold, Roche, & Cuthbert, 2014).

### 4.3 Effects of rearing conditions on recruitment age

The effect of territory or year on the age of recruitment seems to be weak. Indeed, pair formation in Bonelli’s eagles mostly occurs when individuals occupy vacancies left by territorial birds who have died (Hernández‐Matías, Real & Pradel, 2011)—in these cases, a recruiting bird mates with the remaining territorial bird—or, more rarely, when a recruiting bird expels the former territorial bird (unpublished data). This means that Bonelli’s eagles rarely recruit into a new territory. Under this scenario, the recruitment of nonadult individuals could be enhanced by a sudden rise in adult mortality, which will increase the probability that juvenile floaters find and/or occupy a vacant territory or mate with the remaining owner of the territory (Carrascal & Seoane, 2009; Carrete et al., 2006; Penteriani et al., 2003). This phenomenon may explain why males recruit at a younger age than female Bonelli’s eagles, as mortality rates are higher in males than in females in the studied population (Hernández‐Matías, Real, Pradel, Ravayrol, et al., 2011). As a consequence, there is an increase in vacant territories for males who can thus recruit at an earlier age (see also del Mar Delgado et al., 2010).

If low‐quality territories are more easily accessible to young individuals, there may be a trade‐off between recruiting early and trying to recruit into a good‐quality territory (Grande et al., 2009; Kokko & Sutherland, 1998). Our data support the early‐breeding hypothesis, that is to say, that individuals in better body condition—that also may be individuals of superior quality—are more successful when competing for a vacancy and therefore recruit earlier (Figure 6). By recruiting earlier, individuals benefit from having the opportunity to begin their breeding careers sooner and thus improve their fitness (Acker et al., 2018; Mcgraw et al., 1996; Oli et al., 2002). Birds may also gain more experience with their mate and/or become more familiar with the features of their territory (Beletsky & Orians, 1991; Bradley et al., 1990), knowledge that is also thought to improve their breeding performance over the years (Hernández‐Matías, Real, Pradel, Ravayrol, et al., 2011).

### 4.4 Effects of rearing conditions on natal dispersal distance

Natal dispersal distance is determined by both individual and environmental factors interacting in complex fashions (Clobert et al., 2012). In our analyses, both the effect of territory and the year of birth on the natal dispersal distance appeared to be weak, thereby suggesting that the latent variables associated with the year and territory of birth do not have a relevant effect on NDD. On the other hand, effects related to the characteristics of individuals either during their development (i.e., body condition) or at the time of recruitment (i.e., sex and age) did show a detectable signal. As is expected in birds, females had greater NDD than males, (Greenwood & Harvey, 1982; del Mar Delgado et al., 2010; Muriel et al., 2016; Serrano et al., 2003; Soutullo et al., 2006), a finding that coincides with previous studies of this species (Hernández‐Matías et al., 2010). Nevertheless, determinants of NDD associated with individual characteristics appear to interact in a complex way.

The fitted linear models did not reveal any meaningful pattern in males; in which the null model explained as much variance as models containing the studied explanatory variables (i.e., age of recruitment, past productivity of the natal territory, and body condition), thereby suggesting that none of the most relevant variables determining the NDD were accounted for in our analyses. Nevertheless, a graphical representation of the data (Figure 2) suggests that younger birds tend to recruit at shorter NDD than older ones, a pattern that is not followed by the few individuals that recruited during their first calendar year. These birds dispersed over greater distances, a finding that is not, however, reflected in the statistical analyses.

By contrast, younger females recruited at greater NDD than older ones. In the case of young females (3 year old and younger), the NDD had a clear positive relationship with body condition, a pattern not observed in older females. In fact, if dispersal is fueled by stored energy, a disperser with large energy stores (good body condition) might be able to encounter, visit, and sample a larger number of potential habitats before running out of energy than a disperser with low energy resources (Hardouin et al., 2012; Stamps, 2006; Tilgar et al., 2010). This has been reported in ground squirrels *Spermophilus beldingi* (Holekamp & Sherman, 1989), eagle owls *Bubo bubo* (del Mar Delgado et al., 2010), greater flamingos *Phoenicopterus ruber roseus* (Barbraud et al., 2003), and Spanish Imperial eagles *Aquila adalberti* (Soutullo et al., 2013). As well, as explained above, our data supports the early‐breeding hypothesis that states that birds in good body condition will recruit at younger ages than those in poor body condition. Thus, as these healthier individuals recruit when younger, only older females in poorer body condition are left to compete for recruitment and so the correlation between body condition and NDD is lost.

It cannot be excluded the possibility that density played a role in the observed patterns of NDD. In this sense, it is known that density on both the natal and recruitment territories may be important in the natal dispersal process. Indeed, there is an extensive theoretical framework on this issue in which density has been considered a driver that might both favor long‐distance dispersal through increased competition in natal areas; or short‐distance dispersal in the most dense areas either because they are richer in resources or because individuals are attracted to conspecifics (Clobert et al., 2012; Matthysen, 2005). In all cases, it is generally assumed that the most competitive individuals (the ones in better body condition) are less sensitive to high densities and more able to follow the preferred strategy. Therefore, the pattern observed in females—in which the ones with a good body condition recruited at greater distances—would fit with the idea that young females prefer dispersing farther to avoid competition in natal areas. Otherwise, it might be that areas prospected by younger females are placed farther from the natal territory than those prospected by older ones; so, only those young females in good body condition are able to recruit in these territories. Addressing these hypotheses, though, is beyond the scope of the present study, and it would possibly require considering an area spanning a wider range of densities, such as the population of Southern France.

### 4.5 Implications for conservation

Aside from the interest of our findings as further basic knowledge of ecology, it is worth emphasizing the fact that our results have highly relevant implications for management and conservation. Although understanding how animals disperse is key in the correct management and conservation of spatially structured populations (Soutullo et al., 2013), conservation managers typically do not pay enough attention to dispersal processes.

Our study species is threatened in Europe, and considerable conservation efforts are being undertaken to protect its remaining populations (Cadahía et al., 2010; Carrascal & Seoane, 2009; Hernández‐Matías et al., 2015; Real & Mañosa, 2001). This means that managers will need further scientific knowledge of dispersal processes as a tool for implementing conservation actions more efficiently. Restoring the breeding parameters of a target territory or population may be necessary for guaranteeing its mid‐ or long‐term persistence. However, our results reveal that the conditions under which chicks are reared could be relevant too as chicks reared under poor conditions will have less chances to survive until recruitment and thus to contribute to future generations. In this sense, it is worthwhile locating and targeting conservation efforts on the territories with the highest productivity rates (Sergio et al., 2009). In addition, it is vital to improve habitat and prey availability (Ferrer et al., 2014; Real, Bosch, Tinro, & Hernández‐Matías, 2016) and/or to reduce disturbances caused by human outdoor activities and/or the construction of new infrastructures in those territories where chicks are reared under poor conditions (Bosch, Real, Tintó, Zozaya, & Castell, 2010). In all cases, conservation efforts would be best invested in areas of high potential environmental suitability (Carrascal & Seoane, 2009).

The study and assessment of dispersal areas in management and conservation are vital as such areas may be heavily affected by human disturbance and so may be excluded from typical management plans (Cadahía et al., 2010; Carrascal & Seoane, 2009; Penteriani & Delgado, 2009; Rollan, Hernández‐Matías, & Real, 2016). In fact, reducing juvenile mortality in dispersal areas is a crucial conservation strategy for long‐lived species and may have an important effect on the viability of the reproductive fraction of a population (Fasciolo et al., 2016; Hernández‐Matías et al., 2015). However, as these areas are often poorly known, difficult to detect and located in areas managed by more than one regional administration (each with their own particular jurisdictions), far less effort is generally devoted to their conservation than to breeding territories (Cadahía et al., 2010; Penteriani et al., 2015). So, better knowledge of natal dispersal processes is still needed if effective measures for conserving these dispersal areas are to be applied.

At last, it is important to include the body condition of nestlings in monitoring schemes as this information will help detect disturbances to a territory and/or any impoverishment that may occur therein and also ensure that nestlings reach territorial ages.

To summarize, our results illustrate that rearing conditions have a strong effect on the multiple processes involved in natal dispersal. Our findings also highlight the fact that the determinants of these processes are multifaceted and interact in a complex way, which explains the apparent idiosyncrasy of the multiple studies that address dispersal processes. We believe that it is essential to emphasize the need for further studies based on long‐term monitoring schemes over large areas that include individual marking programs. The information gained from such projects will improve our knowledge of the biological determinants behind the complex processes that shape the dynamics and evolution of wild populations, which, in turn, will provide new possibilities for improving conservation practices.

## CONFLICT OF INTEREST

None declared.

## AUTHORS’ CONTRIBUTIONS

MA, JR, and AHM conceived and designed the work. JR, AHM, and others (see acknowledgements) collected the data. MA, JR, and AHM analyzed and interpreted the data. MA and AHM drafted the article. JR and AHM provided critical revision of the article.
