# Supplementary material for: Effects of rearing conditions on natal dispersal processes in a long‐lived predator bird
Source: Ecol Evol. 2018 Jun 13;8(13):6682–98. doi: 10.1002/ece3.4165 (PMC6053564; doi:10.1002/ece3.4165)
Supplement: Supplementary file 1 [file ECE3-8-6682-s001.docx]

**Table SI1.** Summary of the data results from the annual monitoring of Bonelli's eagles on the Catalonia population used in this study. The number of territories by year where chicks were ringed is provided, as well as the number of ringed nestlings and recruited nestlings for each sex. The mean of the different morphometric measures used on the study is also provided for each year and sex.

| **Year** | **Number of Territories** | **Sex** | **Nestlings ringed** | **Fledgings recruited** | **Body weight (g)** | **Tarsus length (mm)** | **Claw length (mm)** | **7th primary feather legth (mm)** | **Tail feather length (mm)** |
| --- | --- | --- | --- | --- | --- | --- | --- | --- | --- |
| 2008 | 22 | Male | 15 | 3 | 1472.333 | 109.087 | 29.613 | 137.467 | 98.200 |
|  |  | Female | 17 | 5 | 1907.000 | 114.894 | 32.547 | 148.941 | 110.294 |
| 2009 | 17 | Male | 17 | 4 | 1464.882 | 110.821 | 29.390 | 124.941 | 90.824 |
|  |  | Female | 11 | 3 | 1770.636 | 111.887 | 31.588 | 119.545 | 88.818 |
| 2010 | 24 | Male | 18 | 7 | 1452.833 | 110.399 | 29.868 | 126.833 | 89.000 |
|  |  | Female | 19 | 5 | 1801.737 | 112.865 | 32.381 | 124.000 | 87.789 |
| 2011 | 24 | Male | 16 | 3 | 1491.375 | 110.728 | 30.409 | 134.563 | 95.000 |
|  |  | Female | 25 | 3 | 1823.600 | 113.111 | 32.521 | 130.840 | 94.280 |
| 2012 | 25 | Male | 22 | 5 | 1375.636 | 105.408 | 28.444 | 106.455 | 74.409 |
|  |  | Female | 14 | 2 | 1669.857 | 106.100 | 30.423 | 103.286 | 74.571 |
| 2013 | 22 | Male | 17 | 3 | 1403.471 | 104.698 | 28.866 | 117.059 | 79.765 |
|  |  | Female | 14 | 6 | 1742.714 | 107.979 | 31.706 | 118.286 | 83.857 |
| 2014 | 30 | Male | 19 | 3 | 1385.684 | 106.029 | 29.071 | 118.368 | 82.789 |
|  |  | Female | 28 | 5 | 1764.500 | 110.774 | 31.867 | 116.000 | 81.107 |
| 2015 | 27 | Male | 24 | 3 | 1403.667 | 107.810 | 29.494 | 119.750 | 84.958 |
|  |  | Female | 22 | 2 | 1823.364 | 111.865 | 32.585 | 121.500 | 87.409 |
| 2016 | 27 | Male | 22 | 0 | 1382.000 | 106.346 | 29.152 | 116.273 | 81.182 |
|  |  | Female | 20 | 0 | 1753.000 | 112.474 | 32.193 | 222.550 | 87.250 |
