# Supplementary material for: Effects of rearing conditions on natal dispersal processes in a long‐lived predator bird
Source: Ecol Evol. 2018 Jun 13;8(13):6682–98. doi: 10.1002/ece3.4165 (PMC6053564; doi:10.1002/ece3.4165)
Supplement: Supplementary file 2 [file ECE3-8-6682-s002.docx]

**Table SI2.** Anova of the effects of sex, body size and their interaction on body weight. The Sum of Squares type III (SS type III) the degrees of liberty (dl), root mean square (RMS) and the F and p values are also shown; as well as the R^2^ value for the corrected model. n = 340; females = 170 and males =170 respectively.

|  | **SS type III** | **dl** | **RMS** | **F** | **p** |
| --- | --- | --- | --- | --- | --- |
| Corrected model | 183.247 | 3 | 61.082 | 132.622 | 0.000 |
| Intercept | 0.000 | 1 | 0.000 | 0.000 | 1.000 |
| Sex | 0.000 | 1 | 0.000 | 0.000 | 1.000 |
| Body size | 183.229 | 1 | 183.229 | 397.827 | 0.000 |
| Sex*Body size | 0.743 | 1 | 0.743 | 1.613 | 0.205 |
| Error | 154.753 | 336 | 0.461 | - | - |
| Total | 338.000 | 340 | - | - | - |
| Corrected total | 338.000 | 339 | - | - | - |

R^2^= 0.542

**Table SI3.** Correlation values of the selected size variables with the body condition index estimated from the residuals of the regression of body size on body mass.

| **Size variables** | **Body Condition** |
| --- | --- |
| Tarsus length | 0.119 |
| Claw length | -0.036 |
| Seventh primary feather length | -0.462 |
| Central tail feather | -0.032 |

**Table SI4.** Effects of territory and year (random factors) on the different variables studied. Model definition shows the factors considered in each model. It is also shown the number of estimable parameters (k), the Akaike's Information Criterion corrected for small sample sizes (AICc), its difference between models (ΔAICc) and the Akaike weights (w_i_) values.

| **Model definition** | **k** | **AICc** | **ΔAICc** | **w_i_** |
| --- | --- | --- | --- | --- |
| **Body condition** |  |  |  |  |
| Intercept + 1\|Territory | 3 | 665.296 | 0.000 | 0.736 |
| Intercept + 1\|Territory + 1\|Year | 4 | 667.344 | 2.048 | 0.264 |
| Intercept | 3 | 689.508 | 24.212 | 0.000 |
| Intercept + 1\|Year | 3 | 691.544 | 26.248 | 0.000 |
| **Recruitment probability** |  |  |  |  |
| Intercept + 1\|Territory + 1\|Year | 3 | 318.675 | 0.000 | 0.663 |
| Intercept + 1\|Territory | 2 | 321.295 | 2.620 | 0.179 |
| Intercept + 1\|Year | 2 | 321.946 | 3.271 | 0.129 |
| Intercept | 2 | 324.973 | 6.298 | 0.028 |
| **Recruitment age** |  |  |  |  |
| Intercept | 3 | 210.924 | 0.000 | 0.555 |
| Intercept + 1\|Territory | 2 | 213.060 | 2.137 | 0.191 |
| Intercept + 1\|Year | 2 | 213.060 | 2.137 | 0.191 |
| Intercept + 1\|Territory + 1\|Year | 2 | 315.271 | 4.347 | 0.063 |

**Table SI4 cont.** Effects of territory and year (random factors) on the different variables studied. Model definition shows the factors considered in each model. It is also shown the number of estimable parameters (k), the Akaike's Information Criterion corrected for small sample sizes (AICc), its difference between models (ΔAICc) and the Akaike weights (w_i_) values.

| **Model definition** | **k** | **AICc** | **ΔAICc** | **w_i_** |
| --- | --- | --- | --- | --- |
| **NDD male** |  |  |  |  |
| Intercept | 3 | 100.747 | 0.000 | 0.601 |
| Intercept + 1\|Territory | 3 | 103.196 | 2.449 | 0.176 |
| Intercept + 1\|Year | 3 | 103.207 | 2.460 | 0.176 |
| Intercept + 1\|Territory + 1\|Year | 4 | 105.845 | 5.099 | 0.047 |
| **NDD female** |  |  |  |  |
| Intercept | 3 | 71.999 | 0.000 | 0.381 |
| Intercept + 1\|Territory | 3 | 72.173 | 0.174 | 0.349 |
| Intercept + 1\|Year | 3 | 73.530 | 1.531 | 0.177 |
| Intercept + 1\|Territory + 1\|Year | 4 | 74.817 | 2.818 | 0.093 |

**Table SI5.** Effects of sex, age of recruitment and their interaction on the logarithm of the natal dispersal distance (NDD). Model definition shows the factors considered in each model. Also given are the number of estimable parameters (k), the Akaike's Information Criterion corrected for small sample sizes (AICc), the difference between models (ΔAICc) and the values of Akaike weights (wi). The effects of territory and year were accounted for in all models by considering them as random factors.

| **Model definition** | **k** | **AICc** | **ΔAICc** | **w_i_** |
| --- | --- | --- | --- | --- |
| Intercept + Sex | 5 | 107.923 | 0.000 | 0.524 |
| Intercept + Sex + Age_Recruitment | 6 | 109.208 | 1.284 | 0.372 |
| Intercept | 4 | 111.342 | 3.804 | 0.000 |
| Intercept + Sex + Age_Recruitment + Sex*Age_Recruitment | 7 | 111.727 | 3.804 | 0.104 |
| Intercept + Age_Recruitmen | 5 | 113.824 | 5.901 | 0.000 |

**SI6. Effects of rearing conditions on natal dispersal distance**

In this analysis, lnNDD (dependent variable) was modelled as a normal response variable using the identity link function and assuming the error to be normally distributed (n=62). We considered age of recruitment, body condition, past productivity, sex and the interaction between age of recruitment with body condition and sex (respectively) as explanatory variables.

Three models including sex, age of recruitment and body condition as explanatory variables were selected from the 64 evaluated models (Table SI5.1). Sex and body condition had a positive coefficient (coefficient=1.20; SE=0.25 and coefficient=0.19; SE=0.18, respectively), unlike age of recruitment, which had a negative coefficient (Coefficient=-0.14; SE=0.10). Based on the relative importance parameter, sex was the most important predictor, followed by age of recruitment and body condition (Table SI5.2). Nevertheless, this last variable was the only one not to contain the zero value in its confidence interval.

Thus, the analysis shows that almost all the variance on NDD is captured by the effect of sex. Consequently, the effects of rearing conditions stay covered up by the effect of sex. An alternative to analyse the effect of rearing conditions on NDD would be to separate the sexes in different data sets in order to remove the effect of sex.

**Table SI6.1** Whole set of models fitted to test the effect of rearing conditions on natal dispersal distance (NDD) for both male and female Bonelli's eagles. Model definition shows the factors considered in each model. Also given are the number of estimable parameters (k), the Akaike's Information Criterion corrected for small sample sizes (AICc), the difference between models (ΔAICc) and the values of Akaike weights (wi). The effects of territory and year were accounted for in all models by considering them as random factors.

| **Model definition** | **k** | **AICc** | **ΔAICc** | **w_i_** |
| --- | --- | --- | --- | --- |
| Intercept + Sex | 5 | 180,955 | 0,000 | 0,152 |
| Intercept + Age_Recruitment + Sex | 6 | 181,637 | 0,683 | 0,108 |
| Intercept + Body_Condition + Sex | 6 | 182,339 | 1,384 | 0,076 |
| Intercept + Sex + Past_Productivity | 6 | 183,221 | 2,266 | 0,049 |
| Intercept + Age_Recruitment + Body_Condition + Sex + Age_Recruitment*Body_Condition | 8 | 183,710 | 2,755 | 0,038 |
| Intercept + Sex + Age_Recruitment*Body_Condition | 6 | 183,710 | 2,755 | 0,038 |
| Intercept + Body_Condition + Sex + Age_Recruitment*Body_Condition | 7 | 183,710 | 2,755 | 0,038 |
| Intercept + Age_Recruitment + Sex + Age_Recruitment*Body_Condition | 7 | 183,710 | 2,755 | 0,038 |
| Intercept + Age_Recruitment + Body_Condition + Sex | 7 | 183,727 | 2,773 | 0,038 |
| Intercept + Age_Recruitment + Sex + Past_Productivity | 7 | 184,138 | 3,183 | 0,031 |

**Table SI6.1 cont.** Whole set of models fitted to test the effect of rearing conditions on natal dispersal distance (NDD) for both male and female Bonelli's eagles. Model definition shows the factors considered in each model. Also given are the number of estimable parameters (k), the Akaike's Information Criterion corrected for small sample sizes (AICc), the difference between models (ΔAICc) and the values of Akaike weights (wi). The effects of territory and year were accounted for in all models by considering them as random factors.

| **Model definition** | **k** | **AICc** | **ΔAICc** | **w_i_** |
| --- | --- | --- | --- | --- |
| Intercept + Sex + Age_Recruitment*Sex | 6 | 184,183 | 3,228 | 0,030 |
| Intercept + Age_Recruitment + Age_Recruitment*Sex | 6 | 184,183 | 3,228 | 0,030 |
| Intercept + Age_Recruitment*Sex | 5 | 184,183 | 3,228 | 0,030 |
| Intercept + Age_Recruitment + Sex + Age_Recruitment*Sex | 7 | 184,183 | 3,228 | 0,030 |
| Intercept + Body_Condition + Sex + Past_Productivity | 7 | 184,882 | 3,927 | 0,021 |
| Intercept + Body_Condition + Age_Recruitment*Sex + Age_Recruitment*Body_Condition | 7 | 186,042 | 5,087 | 0,012 |
| Intercept + Sex + Age_Recruitment*Sex + Age_Recruitment*Body_Condition | 7 | 186,042 | 5,087 | 0,012 |
| Intercept + Age_Recruitment + Age_Recruitment*Sex + Age_Recruitment*Body_Condition | 7 | 186,042 | 5,087 | 0,012 |
| Intercept + Age_Recruitment*Sex + Age_Recruitment*Body_Condition | 6 | 186,042 | 5,087 | 0,012 |
| Intercept + Age_Recruitment + Sex + Age_Recruitment*Sex + Age_Recruitment*Body_Condition | 8 | 186,042 | 5,087 | 0,012 |
| Intercept + Age_Recruitment + Body_Condition + Sex + Age_Recruitment*Sex + Age_Recruitment*Body_Condition | 9 | 186,042 | 5,087 | 0,012 |
| Intercept + Age_Recruitment + Body_Condition + Age_Recruitment*Sex + Age_Recruitment*Body_Condition | 8 | 186,042 | 5,087 | 0,012 |
| Intercept + Body_Condition + Sex + Age_Recruitment*Sex + Age_Recruitment*Body_Condition | 8 | 186,042 | 5,087 | 0,012 |
| Intercept + Age_Recruitment + Body_Condition + Sex + Age_Recruitment*Sex | 8 | 186,351 | 5,396 | 0,010 |
| Intercept + Body_Condition + Age_Recruitment*Sex | 6 | 186,351 | 5,396 | 0,010 |
| Intercept + Age_Recruitment + Body_Condition + Age_Recruitment*Sex | 7 | 186,351 | 5,396 | 0,010 |
| Intercept + Body_Condition + Sex + Age_Recruitment*Sex | 7 | 186,351 | 5,396 | 0,010 |

**Table SI6.1 cont.** Whole set of models fitted to test the effect of rearing conditions on natal dispersal distance (NDD) for both male and female Bonelli's eagles. Model definition shows the factors considered in each model. Also given are the number of estimable parameters (k), the Akaike's Information Criterion corrected for small sample sizes (AICc), the difference between models (ΔAICc) and the values of Akaike weights (wi). The effects of territory and year were accounted for in all models by considering them as random factors.

| **Model definition** | **k** | **AICc** | **ΔAICc** | **w_i_** |
| --- | --- | --- | --- | --- |
| Intercept + Age_Recruitment + Body_Condition + Sex + Past_Productivity | 8 | 186,370 | 5,415 | 0,010 |
| Intercept + Age_Recruitment + Body_Condition + Sex + Age_Recruitemnt*Body_Condition + Past_Productivity | 9 | 186,448 | 5,493 | 0,010 |
| Intercept + Sex + Age_Recruitment*Body_Condition + Past_Productivity | 7 | 186,448 | 5,493 | 0,010 |
| Intercept + Age_Recruitment + Sex + Age_Recruitment*Body_Condition + Past_Productivity | 8 | 186,448 | 5,493 | 0,010 |
| Intercept + Body_Condition + Sex + Age_Recruitment*Body_Condition + Past_Productivity | 8 | 186,448 | 5,493 | 0,010 |
| Intercept + Sex + Age_Recruitment*Sex + Past_Productivity | 7 | 186,776 | 5,821 | 0,008 |
| Intercept + Age_Recruitment + Age_Recruitment*Sex + Past_Productivity | 7 | 186,776 | 5,821 | 0,008 |
| Intercept + Age_Recruitment*Sex + Past_Productivity | 6 | 186,776 | 5,821 | 0,008 |
| Intercept + Age_Recruitment + Sex + Age_Recruitment*Sex + Past_Productivity | 8 | 186,776 | 5,821 | 0,008 |
| Intercept + Body_Condition + Age_Recruitment*Sex + Age_Recruitment*Body_Condition + Past_Productivity | 8 | 188,894 | 7,939 | 0,003 |
| Intercept + Sex + Age_Recruitment*Sex + Age_Recruitment*Body_Condition + Past_Productivity | 8 | 188,894 | 7,939 | 0,003 |
| Intercept + Age_Recruitment + Body_Condition + Sex + Age_Recruitment*Sex + Age_Recruitment*Body_Condition + Past_Productivity | 9 | 188,894 | 7,939 | 0,003 |
| Intercept + Age_Recruitment + Body_Condition + Age_Recruitment*Sex + Age_Recruitment*Body_Condition + Past_Productivity | 9 | 188,894 | 7,939 | 0,003 |
| Intercept + Body_Condition + Sex + Age_Recruitment*Sex + Age_Recruitment*Body_Condition + Past_Productivity | 9 | 188,894 | 7,939 | 0,003 |

**Table SI6.1 cont.** Whole set of models fitted to test the effect of rearing conditions on natal dispersal distance (NDD) for both male and female Bonelli's eagles. Model definition shows the factors considered in each model. Also given are the number of estimable parameters (k), the Akaike's Information Criterion corrected for small sample sizes (AICc), the difference between models (ΔAICc) and the values of Akaike weights (wi). The effects of territory and year were accounted for in all models by considering them as random factors.

| **Model definition** | **k** | **AICc** | **ΔAICc** | **w_i_** |
| --- | --- | --- | --- | --- |
| Intercept + Age_Recruitment + Age_Recruitment*Sex + Age_Recruitment*Body_Condition + Past_Productivity | 8 | 188,894 | 7,939 | 0,003 |
| Intercept + Age_Recruitment*Sex + Age_Recruitment*Body_Condition + Past_Productivity | 7 | 188,894 | 7,939 | 0,003 |
| Intercept + Age_Recruitment + Sex + Age_Recruitment*Sex + Age_Recruitment*Body_Condition + Past_Productivity | 9 | 188,894 | 7,939 | 0,003 |
| Intercept + Body_Condition + Age_Recruitment*Sex + Past_Productivity | 7 | 189,096 | 8,141 | 0,003 |
| Intercept + Age_Recruitment + Body_Condition + Sex + Age_Recruitment*Sex + Past_Productivity | 8 | 189,096 | 8,141 | 0,003 |
| Intercept + Age_Recruitment + Body_Condition + Age_Recruitment*Sex + Past_Productivity | 8 | 189,096 | 8,141 | 0,003 |
| Intercept + Body_Condition + Sex + Age_Recruitment*Sex + Past_Productivity | 8 | 189,096 | 8,141 | 0,003 |
| Intercept | 4 | 197,750 | 16,795 | 0,000 |
| Intercept + Body_Condition | 5 | 199,874 | 18,919 | 0,000 |
| Intercept + Past_Productivity | 5 | 199,932 | 18,977 | 0,000 |
| Intercept + Age_Recruitment | 5 | 200,119 | 19,164 | 0,000 |
| Intercept + Body_Condition + Past_Productivity | 6 | 201,845 | 20,890 | 0,000 |
| Intercept + Age_Recruitment + Body_Condition | 6 | 202,296 | 21,342 | 0,000 |
| Intercept + Age_Recruitment + Past_Productivity | 6 | 202,382 | 21,427 | 0,000 |
| Intercept + Age_Recruitment + Age_Recruitment*Body_Condition | 6 | 203,998 | 23,043 | 0,000 |
| Intercept + Body_Condition + Age_Recruitment*Body_Condition | 6 | 203,998 | 23,043 | 0,000 |
| Intercept + Age_Recruitment*Body_Condition | 5 | 203,998 | 23,043 | 0,000 |
| Intercept + Age_Recruitment + Body_Condition + Age_Recruitment*Body_Condition | 7 | 203,998 | 23,043 | 0,000 |

**Table SI6.1 cont.** Whole set of models fitted to test the effect of rearing conditions on natal dispersal distance (NDD) for both male and female Bonelli's eagles. Model definition shows the factors considered in each model. Also given are the number of estimable parameters (k), the Akaike's Information Criterion corrected for small sample sizes (AICc), the difference between models (ΔAICc) and the values of Akaike weights (wi). The effects of territory and year were accounted for in all models by considering them as random factors.

| **Model definition** | **k** | **AICc** | **ΔAICc** | **w_i_** |
| --- | --- | --- | --- | --- |
| Intercept + Age_Recruitment + Body_Condition + Past_Productivity | 7 | 204,384 | 23,429 | 0,000 |
| Intercept + Age_Recruitment + Age_Recruitment*Body_Condition + Past_Productivity | 7 | 206,049 | 25,094 | 0,000 |
| Intercept + Age_Recruitment*Body_Condition + Past_Productivity | 6 | 206,049 | 25,094 | 0,000 |
| Intercept + Age_Recruitment + Body_Condition + Age_Recruitment*Body_Condition + Past_Productivity | 8 | 206,049 | 25,094 | 0,000 |
| Intercept + Body_Condition + Age_Recruitment*Body_Condition + Past_Productivity | 7 | 206,049 | 25,094 | 0,000 |

**Table SI6.2** Effects on NDD of the parameters selected from the best-fitting models (ΔAICc<2) after model averaging. The relative importance of considered variables expresses the sum of the Akaike weights of the models containing the parameter in question. The effects of territory and year were accounted for in all models by considering them as random factors.

| **Parameter** | **Estimate** | **SE** | **95% Confidence interval** | **Relative importance** |
| --- | --- | --- | --- | --- |
| Intercept | 3.573 | 0.299 | (2.978, 4.168) |  |
| Sex | 1.197 | 0.249 | (0.699, 1.695) | 1.00 |
| Age_Recruitment | -0.140 | 0.104 | (-0.348, 0.069) | 0.32 |
| Body_Condition | 0.187 | 0.178 | (-0.169, 0.543) | 0.23 |

**Table SI7.** Whole set of models fitted to test the effect of rearing conditions in the body condition of the nestlings. Model definition shows the factors considered in each model. Also given are the number of estimable parameters (k), the Akaike's Information Criterion corrected for small sample sizes (AICc), the difference between models (ΔAICc) and the values of Akaike weights (wi). The effects of territory and year were accounted for in all models by considering them as random factors.

| **Model definition** | **k** | **AICc** | **ΔAICc** | **w_i_** |
| --- | --- | --- | --- | --- |
| Intercept + Past_Productivity | 5 | 663.785 | 0.000 | 0.464 |
| Intercept + Past_Productivity + Num_Nestlings | 6 | 665.715 | 1.930 | 0.177 |
| Intercept + 1\|Territory + 1\|Year | 4 | 667.344 | 3.559 | 0.078 |
| Intercept + Past_Productivity + Num_Nestlings + Past_Productivity*Num_Nestlings | 7 | 667.780 | 3.995 | 0.063 |
| Intercept + Past_Productivity* Num_Nestlings | 5 | 667.780 | 3.995 | 0.063 |
| Intercept + Past_Productivity + Past_Productivity*Num_Nestlings | 6 | 667.780 | 3.995 | 0.063 |
| Intercept + Num_Nestlings + Past_Productivity*Num_Nestlings | 6 | 667.780 | 3.995 | 0.063 |
| Intercept + Num_Nestlings | 5 | 669.367 | 5.582 | 0.028 |

**Table SI8.** Whole set of models fitted to test the effect of rearing conditions on the probability of survival until recruitment. Model definition shows the factors considered in each model. Also given are the number of estimable parameters (k), the Akaike's Information Criterion corrected for small sample sizes (AICc), the difference between models (ΔAICc) and the values of Akaike weights (wi). The effects of territory and year were accounted for in all models by considering them as random factors.

| **Model definition** | **k** | **AICc** | **ΔAICc** | **w_i_** |
| --- | --- | --- | --- | --- |
| Intercept + Body_Condition + Age | 5 | 314.147 | 0.000 | 0.337 |
| Intercept + Age | 4 | 314.666 | 0.519 | 0.260 |
| Intercept + Body_Condition + Past_Productivity + Age | 6 | 315.821 | 1.674 | 0.146 |
| Intercept + Past_Productivity + Age | 5 | 315.918 | 1.771 | 0.139 |
| Intercept + Body_Condition | 4 | 318.361 | 4.214 | 0.041 |
| Intercept | 3 | 318.675 | 4.528 | 0.035 |
| Intercept + Past_Productivity | 4 | 319.643 | 5.496 | 0.022 |
| Intercept + Body_Condition + Past_Productivity | 5 | 319.779 | 5.632 | 0.020 |

**Table SI9**. Whole set of models fitted to test the effect of rearing conditions on the age of recruitment. Model definition shows the factors considered in each model. Also given are the number of estimable parameters (k), the Akaike's Information Criterion corrected for small sample sizes (AICc), the difference between models (ΔAICc) and the values of Akaike weights (wi). The effects of territory and year were accounted for in all models by considering them as random factors.

| **Model definition** | **k** | **AICc** | **ΔAICc** | **w_i_** |
| --- | --- | --- | --- | --- |
| Intercept + Body_Condition | 4 | 213.716 | 0.000 | 0.126 |
| Intercept + Body_Condition + Sex | 5 | 214.237 | 0.522 | 0.097 |
| Intercept + Sex | 4 | 215.144 | 1.428 | 0.062 |
| Intercept | 3 | 215.271 | 1.555 | 0.058 |
| Intercept + Past_Productivity | 4 | 215.365 | 1.649 | 0.055 |
| Intercept + Body_Condition + Past_Productivity | 5 | 215.610 | 1.894 | 0.049 |
| Intercept + Past_Productivity + Sex | 6 | 216.098 | 2.382 | 0.038 |
| Intercept + Sex + Body_Condition*Sex | 6 | 216.239 | 2.523 | 0.036 |
| Intercept + Body_Condition + Body_Condition*Sex | 6 | 216.239 | 2.523 | 0.036 |
| Intercept + Body_Condition*Sex | 5 | 216.239 | 2.523 | 0.036 |
| Intercept + Body_Condition + Sex + Body_Condition*Sex | 7 | 216.239 | 2.523 | 0.036 |
| Intercept + Body_Condition + Past_Productivity + Sex | 7 | 216.472 | 2.756 | 0.032 |
| Intercept + Body_Condition + Body_Condition*Past_Productivity | 6 | 217.827 | 4.111 | 0.016 |
| Intercept + Past_Productivity + Body_Condition*Past_Productivity | 6 | 217.827 | 4.111 | 0.016 |
| Intercept + Body_Condition*Past_Productivity | 5 | 217.827 | 4.111 | 0.016 |
| Intercept + Body_Condition + Past_Productivity + Body_Condition*Past_Productivity | 7 | 217.827 | 4.111 | 0.016 |
| Intercept + Sex + Past_Productivity*Sex | 6 | 218.295 | 4.579 | 0.013 |
| Intercept + Past_Productivity + Past_Productivity*Sex | 6 | 218.295 | 4.579 | 0.013 |
| Intercept + Past_Productivity*Sex | 5 | 218.295 | 4.579 | 0.013 |
| Intercept + Past_Productivity + Sex + Past_Productivity*Sex | 7 | 218.295 | 4.579 | 0.013 |
| Intercept + Body_Condition + Past_Productivity + Sex + Body_Condition*Past_Productivity | 8 | 218.493 | 4.777 | 0.012 |
| Intercept + Sex + Body_Condition*Past_Productivity | 6 | 218.493 | 4.777 | 0.012 |

**Table SI9 cont.** Whole set of models fitted to test the effect of rearing conditions on the age of recruitment. Model definition shows the factors considered in each model. Also given are the number of estimable parameters (k), the Akaike's Information Criterion corrected for small sample sizes (AICc), the difference between models (ΔAICc) and the values of Akaike weights (wi). The effects of territory and year were accounted for in all models by considering them as random factors.

| **Model definition** | **k** | **AICc** | **ΔAICc** | **w_i_** |
| --- | --- | --- | --- | --- |
| Intercept + Body_Condition + Sex + Body_Condition*Past_Productivity | 7 | 218.493 | 4.777 | 0.012 |
| Intercept + Past_Productivity + Sex + Body_Condition*Past_Productivity | 7 | 218.493 | 4.777 | 0.012 |
| Intercept + Body_Condition + Past_Productivity + Sex + Body_Condition*Sex | 8 | 218.515 | 4.799 | 0.011 |
| Intercept + Past_Productivity + Body_Condition*Sex | 6 | 218.515 | 4.799 | 0.011 |
| Intercept + Body_Condition + Past_Productivity + Body_Condition*Sex | 7 | 218.515 | 4.799 | 0.011 |
| Intercept + Past_Productivity + Sex + Body_Condition*Sex | 7 | 218.515 | 4.799 | 0.011 |
| Intercept + Body_Condition + Past_Productivity + Sex + Past_Productivity*Sex | 8 | 218.974 | 5.258 | 0.009 |
| Intercept + Body_Condition + Past_Productivity*Sex | 6 | 218.974 | 5.258 | 0.009 |
| Intercept + Body_Condition + Past_Productivity + Past_Productivity*Sex | 7 | 218.974 | 5.258 | 0.009 |
| Intercept + Body_Condition + Sex + Past_Productivity*Sex | 7 | 218.974 | 5.258 | 0.009 |
| Intercept + Body_Condition + Past_Productivity + Sex + Body_Condition*Sex + Body_Condition*Past_Productivity | 8 | 220.674 | 6.958 | 0.004 |
| Intercept + Sex + Body_Condition*Sex + Body_Condition*Past_Productivity | 7 | 220.674 | 6.958 | 0.004 |
| Intercept + Body_Condition + Body_Condition*Sex + Body_Condition*Past_Productivity | 7 | 220.674 | 6.958 | 0.004 |
| Intercept + Past_Productivity + Body_Condition*Sex + Body_Condition*Past_Productivity | 7 | 220.674 | 6.958 | 0.004 |
| Intercept + Body_Condition*Sex + Body_Condition*Past_Productivity | 6 | 220.674 | 6.958 | 0.004 |

**Table SI9 cont.** Whole set of models fitted to test the effect of rearing conditions on the age of recruitment. Model definition shows the factors considered in each model. Also given are the number of estimable parameters (k), the Akaike's Information Criterion corrected for small sample sizes (AICc), the difference between models (ΔAICc) and the values of Akaike weights (wi). The effects of territory and year were accounted for in all models by considering them as random factors.

| **Model definition** | **k** | **AICc** | **ΔAICc** | **w_i_** |
| --- | --- | --- | --- | --- |
| Intercept + Body_Condition + Past_Productivity + Body_Condition*Sex + Body_Condition*Past_Productivity | 8 | 220.674 | 6.958 | 0.004 |
| Intercept + Body_Condition + Sex + Body_Condition*Sex + Body_Condition*Past_Productivity | 8 | 220.674 | 6.958 | 0.004 |
| Intercept + Past_Productivity + Sex + Body_Condition*Sex + Body_Condition*Past_Productivity | 8 | 220.674 | 6.958 | 0.004 |
| Intercept + Body_Condition + Past_Productivity + Sex + Body_Condition*Sex + Past_Productivity_Sex | 9 | 220.820 | 7.104 | 0.004 |
| Intercept + Sex + Body_Condition*Sex + Past_Productivity*Sex | 7 | 220.820 | 7.104 | 0.004 |
| Intercept + Body_Condition + Body_Condition*Sex + Past_Productivity*Sex | 7 | 220.820 | 7.104 | 0.004 |
| Intercept + Past_Productivity + Body_Condition*Sex + Past_Productivity*Sex | 7 | 220.820 | 7.104 | 0.004 |
| Intercept + Body_Condition*Sex + Past_Productivity*Sex | 6 | 220.820 | 7.104 | 0.004 |
| Intercept + Body_Condition + Past_Productivity + Body_Condition*Sex + Past_Productivity*Sex | 8 | 220.820 | 7.104 | 0.004 |
| Intercept + Body_Condition + Sex + Body_Condition*Sex + Past_Productivity*Sex | 8 | 220.820 | 7.104 | 0.004 |
| Intercept + Past_Productivity + Sex + Body_Condition*Sex + Past_Productivity*Sex | 8 | 220.820 | 7.104 | 0.004 |
| Intercept + Body_Condition + Past_Productivity + Sex + Body_Condition*Past_Productivity + Past_Productivity*Sex | 9 | 221.104 | 7.388 | 0.003 |

**Table SI9 cont.** Whole set of models fitted to test the effect of rearing conditions on the age of recruitment. Model definition shows the factors considered in each model. Also given are the number of estimable parameters (k), the Akaike's Information Criterion corrected for small sample sizes (AICc), the difference between models (ΔAICc) and the values of Akaike weights (wi). The effects of territory and year were accounted for in all models by considering them as random factors.

| **Model definition** | **k** | **AICc** | **ΔAICc** | **w_i_** |
| --- | --- | --- | --- | --- |
| Intercept + Sex + Body_Condition*Past_Productivity + Past_Productivity*Sex | 7 | 221.104 | 7.388 | 0.003 |
| Intercept + Body_Condition + Body_Condition*Past_Productivity + Past_Productivity*Sex | 7 | 221.104 | 7.388 | 0.003 |
| Intercept + Past_Productivity + Body_Condition*Past_Productivity + Past_Productivity*Sex | 7 | 221.104 | 7.388 | 0.003 |
| Intercept + Body_Condition*Past_Productivity + Past_Productivity*Sex | 6 | 221.104 | 7.388 | 0.003 |
| Intercept + Body_Condition + Past_Productivity + Body_Condition*Past_Productivity + Past_Productivity*Sex | 8 | 221.104 | 7.388 | 0.003 |
| Intercept + Body_Condition + Sex + Body_Condition*Past_Productivity + Past_Productivity*Sex | 8 | 221.104 | 7.388 | 0.003 |
| Intercept + Past_productivity + Sex + Body_Condition*Past_Productivity + Past_Productivity*Sex | 8 | 221.104 | 7.388 | 0.003 |
| Intercept + Body_Condition + Past_Productivity + Sex + Body_Condition*Sex + Body_Condition*Past_Productivity + Past_Productivity*Sex | 9 | 223.096 | 9.381 | 0.001 |
| Intercept + Sex + Body_Condition*Sex + Body_Condition*Past_Productivity + Past_Productivity*Sex | 8 | 223.096 | 9.381 | 0.001 |
| Intercept + Body_Condition + Body_Condition*Sex + Body_Condition*Past_Productivity + Past_Productivity*Sex | 8 | 223.096 | 9.381 | 0.001 |

**Table SI9 cont.** Whole set of models fitted to test the effect of rearing conditions on the age of recruitment. Model definition shows the factors considered in each model. Also given are the number of estimable parameters (k), the Akaike's Information Criterion corrected for small sample sizes (AICc), the difference between models (ΔAICc) and the values of Akaike weights (wi). The effects of territory and year were accounted for in all models by considering them as random factors.

| **Model definition** | **k** | **AICc** | **ΔAICc** | **w_i_** |
| --- | --- | --- | --- | --- |
| Intercept + Past_Productivity + Body_Condition*Sex + Body_Condition*Past_Productivity + Past_Productivity*Sex | 8 | 223.096 | 9.381 | 0.001 |
| Intercept + Body_Condition*Sex + Body*Condition*Past_Productivity + Past_Productivity*Sex | 7 | 223.096 | 9.381 | 0.001 |
| Intercept + Body_Condition + Past_Productivity + Body_Condition*Sex + Body_Condition*Past_Productivity + Past_Productivity*Sex | 9 | 223.096 | 9.381 | 0.001 |
| Intercept + Body_Condition + Sex + Body_Condition*Sex + Body_Condition*Past_Productivity + Past_Productivity*Sex | 9 | 223.096 | 9.381 | 0.001 |
| Intercept + Past_Productivity + Sex + Body_Condition*Sex + Body_Condition*Past_Productivity + Past_Productivity*Sex | 9 | 223.096 | 9.381 | 0.001 |

**Table SI10.** Whole set of models fitted to test the effect of rearing conditions on natal dispersal distance (NDD) for male Bonelli's eagles. Model definition shows the factors considered in each model. Also given are the number of estimable parameters (k), the Akaike's Information Criterion corrected for small sample sizes (AICc), the difference between models (ΔAICc) and the values of Akaike weights (wi). The effects of territory and year were accounted for in all models by considering them as random factors.

| **Model definition** | **k** | **AICc** | **ΔAICc** | **w_i_** |
| --- | --- | --- | --- | --- |
| Intercept | 4 | 105.845 | 0.000 | 0.472 |
| Intercept + Age_Recruitment | 5 | 108.242 | 2.397 | 0.142 |
| Intercept + Past_Productivity | 5 | 108.287 | 2.442 | 0.139 |
| Intercept + Body_Condition | 5 | 108.430 | 2.585 | 0.130 |
| Intercept + Age_Recruitment + Past_Productivity | 6 | 111.096 | 5.251 | 0.034 |
| Intercept + Age_Recruitment + Body_Condition | 6 | 111.269 | 5.424 | 0.031 |
| Intercept + Body_Condition + Past_Productivity | 6 | 111.343 | 5.497 | 0.030 |
| Intercept + Age_Recruitment + Body_Condition + Age_Recruitment*Body_Condition | 7 | 113.722 | 7.876 | 0.009 |
| Intercept + Age_Recruitment + Body_Condition + Past_Productivity | 7 | 114.465 | 8.260 | 0.006 |
| Intercept + Body_Condition + Past_Productivity + Age_Recruitment*Body_Condition | 7 | 117.062 | 11.216 | 0.002 |
| Intercept + Age_Recruitment + Past_Productivity + Age_Recruitment*Body_Condition | 7 | 117.062 | 11.216 | 0.002 |
| Intercept + Age_Recruitment + Body_Condition + Past_Productivity + Age_Recruitment*Body_Condition | 8 | 117.062 | 11.216 | 0.002 |

**Table SI11.** Whole set of models fitted to test the effect of rearing conditions on natal dispersal distance (NDD) for female Bonelli's eagles. Model definition shows the factors considered in each model. Also given are the number of estimable parameters (k), the Akaike's Information Criterion corrected for small sample sizes (AICc), the difference between models (ΔAICc) and the values of Akaike weights (wi). The effects of territory and year were accounted for in all models by considering them as random factors.

| **Model definition** | **k** | **AICc** | **ΔAICc** | **w_i_** |
| --- | --- | --- | --- | --- |
| Intercept + Age_Recruitment + Body_Condition + Age_Recruitment*Body_Condition | 7 | 74.370 | 0.000 | 0.236 |
| Intercept | 4 | 74.817 | 0.447 | 0.189 |
| Intercept + Body_Condition | 5 | 75.887 | 1.518 | 0.111 |
| Intercept + Age_Recruitment | 5 | 76.080 | 1.710 | 0.100 |
| Intercept + Age_Recruitment + Body_Condition + Past_Productivity + Age_Recruitment*Body_Condition | 8 | 76.721 | 2.351 | 0.073 |
| Intercept + Body_Condition + Past_Productivity + Age_Recruitment*Body_Condition | 7 | 76.721 | 2.351 | 0.073 |
| Intercept + Age_Recruitment + Past_Productivity + Age_Recruitment*Body_Condition | 7 | 76.721 | 2.351 | 0.073 |
| Intercept + Past_Productivity | 5 | 77.418 | 3.048 | 0.051 |
| Intercept + Body_Condition + Past_Productivity | 6 | 78.331 | 3.962 | 0.033 |
| Intercept + Age_Recruitment + Body_Condition | 6 | 78.497 | 4.128 | 0.030 |
| Intercept + Age_Recruitment + Past_Productivity | 6 | 78.915 | 4.546 | 0.024 |
| Intercept + Age_Recruitment + Body_Condition + Past_Productivity | 7 | 81.199 | 6.829 | 0.008 |
